# Three-dimensional quantitative structure activity relationship (QSAR) of cytotoxic active 3,5-diaryl-4,5-dihydropyrazole analogs: a comparative molecular field analysis (CoMFA) revisited study

**DOI:** 10.1186/1752-153X-6-50

**Published:** 2012-05-30

**Authors:** Abdel-Sattar S Hamad Elgazwy, DaliaH S Soliman, Saad R Atta-Allah, Diaa A Ibrahim

**Affiliations:** 1 Department of Chemistry Faculty of Science, Ain Shams University, Abbassia, 11566, Cairo, Egypt; 2 Department of pharmaceutical chemistry, Faculty of Pharmacy (girls' branch), Al-Azhar University, Nasser city, Cairo, Egypt; 3 National Organization for Drug Control & Research, P.O. Box: 29, Cairo, Egypt

**Keywords:** 3,5-diaryl-4,5-dihydropyrazole-1-carbothioamide, 1-(3,5-diaryl-4,5-dihydropyrazol-1-yl)ethanethione, Antitumor, QSAR, CoMFA

## Abstract

In vitro antitumor evaluation of the synthesized 46 compounds of 3,5-diaryl-4,5-dihydropyrazoles against EAC cell lines and 3D QSAR study using pharmacophore and Comparative Molecular Field Analysis **(**CoMFA) methods were described. CoMFA derived QSAR model shows a good conventional squared correlation coefficient r^2^ and cross validated correlation coefficient r^2^cv 0.896 and 0.568 respectively. In this analysis steric and electrostatic field contribute to the QSAR equation by 70% and 30% respectively, suggesting that variation in biological activity of the compounds is dominated by differences in steric (van der Waals) interactions. To visualize the CoMFA steric and electrostatic field from partial least squares (PLS) analysis, contour maps are plotted as percentage contribution to the QSAR equation and are associated with the differences in biological activity.

**Background:**

Pyrazole derivatives exhibit a wide range of biological properties including promising antitumor activity. Furthermore, Aldol condensation assisted organic synthesis has delivered rapid routes to *N*-containing heterocycles, including pyrazoles. Combining these features, the use of chalconisation-assisted processes will provide rapid access to a targeted dihydropyrazoles library bearing a hydrazino 3D QSAR study using pharmacophore and Comparative Molecular Field Analysis **(**CoMFA) methods were described for evaluation of antioxidant properties.

**Results:**

Chalcones promoted 1 of the 2 steps in a rapid, convergent synthesis of a small library of hydrazinyl pyrazole derivatives, all of which exhibited significant antitumor activity against Ehrlich Ascites Carcinoma (EAC) human tumor cell line comparable to that of the natural anticancer doxorubicin, as a reference standard during this study. In order to understand the observed pharmacological properties, quantitative structure-activity relationship (3D QSAR) study was initiated.

**Conclusions:**

Chalcones heating provides a rapid and expedient route to a series of pyrazoles to investigate their chracterization scavenging properties. Given their favorable properties, in comparison with known anticancer, these pyrazole derivatives are promising leads for further development and optimization.

## Introduction

The cancer chemotherapy has entered a new era of molecularly targeted therapeutics, that highly selective and not associated with the serious toxicities of conventional cytotoxic drugs [1]. The urea and thiourea derivatives played an important role in anticancer agents because of their good inhibitory activity against receptor tyrosine kinases (RTKs), protein tyrosine kinases (PTKs), and NADH oxidase which played also critical roles in many aspects of tumorigenesis [2-4]. Many pyrazole derivatives were found to possess a wide range of bioactivities, such as anti-viral and anti-tumor [5-7].

Much attention was paid to pyrazoles as a potential antimicrobial agent after the discovery of natural pyrazole C-glycoside, pyrazofurin that demonstrated a broad spectrum of antimicrobial activity [8]. To the best of our knowledge, few reports have been dedicated to the synthesis of 3, 5-diaryl-4, 5-dihydropyrazole-1-carbothioamide and 1-(3, 5-diaryl-4, 5-dihydropyrazol-1-yl) ethanone as EAC cells inhibitory activities.

In continuation to anticancer compounds with EAC cells [9], the biological evaluation indicated that some of synthesized compounds are potent inhibitors of cytotoxic activity. The compound **D5** displayed the most potent inhibitory activity with IC_50_ of 0.09 μM, which was comparable to the positive control doxorubicin (IC_50_ = 7.36 μM) as reference drug. In the present work, we report the synthesis of pyrazole derivatives containing thiourea and urea skeletons **D1-30** and **F1-16** with CoMFA study, not only for validating the observed pharmacological properties but also, for investigating the most important parameters controlling these properties as anticancer agents.

## Results and discussion

### Chemistry

The synthetic Pyrazoles are provided to be useful synthetic intermediates for synthesis of biologically active deazafolates ring system and pyrimidine nucleosides, which are reported to be significantly active, both in vitro and in vivo [10,11] as dihydrofolate reductase inhibitors [12]. Their cytotoxicity against various experimental tumors were found to be as potentially methotrexate [13,14], one of the most effective antimetabolites currently used in treatment of various solid tumors [15,16]. Owing to our plane to develop an efficient and simple procedure for the synthesis of new antimetabolites [17,18], in this part of new synthesis and due to the development of an efficient and simple method for synthesis of new antitumor agents for some of 3, 5-diaryl-4, 5-dihydropyrazole-1-carbothioamide **D1-30** and 1-(3, 5-diaryl-4, 5-dihydropyrazol-1-yl)ethanone **F1-16** analogs we presented here this research article. Firstly, Chalcones can be prepared by an aldol condensation between a substituted benzaldehyde and substituted acetophenone in the presence of sodium hydroxide as a catalyst.

This reaction has been found to work without any solvent at all a solid-state reaction [19]. The reaction between substituted benzaldehydes and acetophenones has been used to demonstrate green chemistry in undergraduate chemistry education [20]. In a study investigating green chemistry synthesis, chalcones were also synthesized from the same starting materials in high temperature (200 to 350°C) [21].

**Scheme 1:**
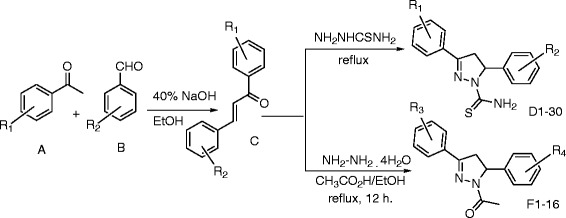
One-step synthesis of chalcones and their use in the synthesis of 3, 5-diaryl-1-substituted-4,5-dihydropyrazoles.

Thus, it has been found that chalcones **C** reacted with thiosemicarbazide to give the corresponding 3, 5-diaryl-4, 5-dihydropyrazole-1-carbothioamide **D1-30** while reacted with hydrazine hydrate in the presence of acetic acid gave 1-(3, 5-diaryl-4, 5-dihydropyrazol-1-yl)ethanone **F1-16** in good yields. The synthesis of compounds **D1–30** and **F1–16** followed the general pathway as outlined in Scheme 1, gave satisfactory analytical and spectroscopic data, which were in full accordance with their depicted structures.

### Anti-tumor properties

The synthesized compounds (**D1-30** and **F1-16**) were screened for their anti-tumor properties against Ehrlich Ascites Carcinoma (EAC) human tumor cell line. From the observed results presented in Tables 1 and Table 2 it has been noticed that most of the synthesized compounds reveal mild to potent anti-tumor properties against (EAC) human tumor cell lines. Meanwhile, compound **D5** exhibit high potent anti-tumor activity with potency (IC_50_, concentration required to produce 50% inhibition of cell growth compared to control experimental) = 0.09 μM, compared with Doxorubicin (IC_50_ = 7.36 μM), which was used as a reference standard during this study. In order to understand the observed pharmacological properties, quantitative structure-activity relationship (3D QSAR) study was initiated.

**Table 1:** Structure and biological activities of training set molecules (D1-30)

**No.**	**Compound**	**IC50 (μM)**	**No.**	**Compound**	**IC50 (μM)**
**D1**		0.88	**D2**		1.87
**D3**		2.39	**D4**		0.54
**D5**		0.09	**D6**		0.19
**D7**		3.09	**D8**		5.28
**D9**		3.99	**D10**		4.44
**D11**		6.64	**D12**		7.54
**D13**		6.77	**D14**		7.68
**D15**		5.88	**D16**		5.33
**D17**		7.89	**D18**		6.66
**D19**		7.32	**D20**		6.56
**D21**		8.99	**D22**		8.26
**D23**		10.09	**D24**		9.88
**D25**		8.19	**D26**		11.24
**D27**		10.77	**D28**		13.48
**D29**		12.35	**D30**		11.09

**Table 2:** Structure and biological activities of test set molecules (F1-16)

**No.**	**Structure**	**IC50 (μM)**	**No.**	**Structure**	**IC50 (μM)**
**F1**		18.45	**F2**		16.16
**F3**		18.47	**F4**		19.34
**F5**		17.21	**F6**		24.21
**F7**		28.71	**F8**		25.52
**F9**		15.61	**F10**		19.74
**F11**		20.64	**F12**		17.52
**F13**		15.34	**F14**		21.48
**F15**		20.06	**F16**		17.33

### QSAR study

#### Pharmacophore modeling

All compounds were built using the Discovery Studio 2.5 software (Accelrys Inc., San Diego, CA, USA), and optimized by using a CHARMm-like force field [22]. The Hip Hop model treats molecular structures as templates consisting of strategically positioned chemical functions that will bind effectively with complementary functions on receptor (Table 1).

The biologically most important binding functions are deduced from a small set of compounds that cover a broad range of activity. In this paper, considering the relatively high flexibility of ligands under investigation, the conformational analyses are carried out with an energy window high enough to include the bioactive conformation.

**Figure 1:**
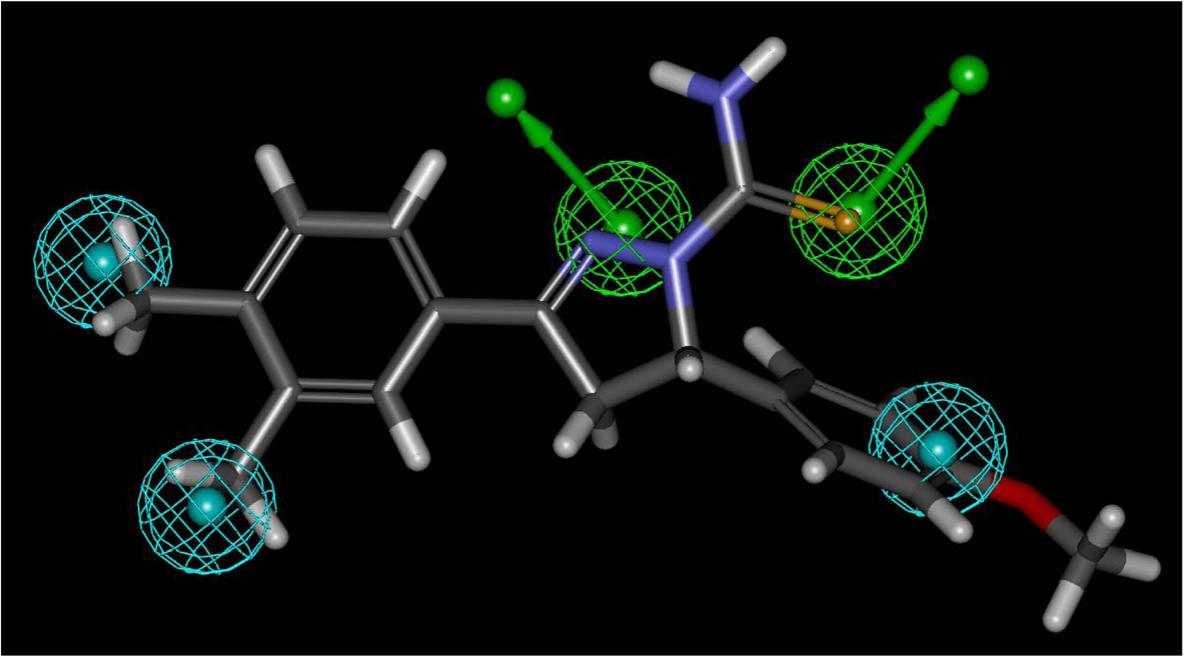
Superimposition of compound D5 with the best pharmacophore model.

DS 2.5 automatically generated conformational models for each compound. Diverse conformational models for each compound were generated such that the conformers covered accessible conformational space defined within 20 kcal/mol of the estimated global minimum. The estimation of conformational energy was performed based on the CHARMm force field. CATALYST provides two types of conformational analysis: fast and best quality. The best option was used, specifying 250 as the maximum number of conformers. Hypotheses approximating the 3D QSAR pharmacophore were described as a set of features distributed within 3D space with the correlation to the biological results. Five kinds of surface-accessible functions were considered, including two hydrogen bond acceptors (HBA) and 3 hydrophobic centers, as shown in Figure 1.

**Figure 2:**
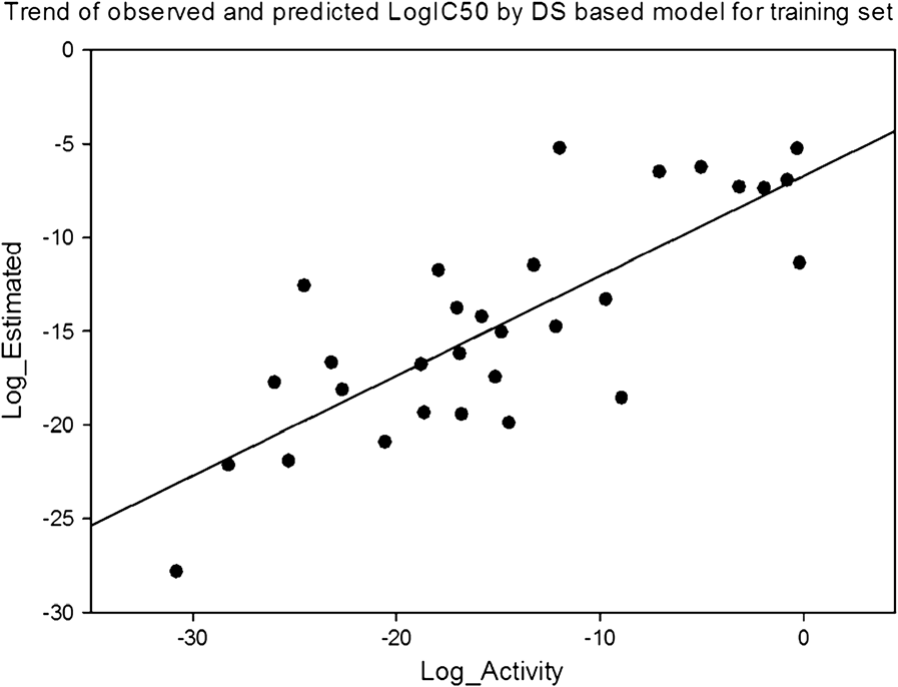
Correlation line of log actual activity vs log estimated activity (r 0.695) of the best pharmacophore model.

Furthermore, to emphasize the importance of an aromatic group of the studied molecules, aromatic ring (AR), which consists of directionality, was also included in the subsequent run. Considering the complexity of the studied molecules, the hypothesis generator was restricted to select only five or less features. During a hypothesis generation run, it attempts to minimize a score function consisting of two terms. One term penalizes the deviation between the estimated activities of the molecules in data set and their experimental values; the other term penalizes the complexity of the hypothesis. After assessing all generated hypotheses, the most plausible one is considered the best, as presented in Table 3 and displayed in Figure 2.

**Table 3:** Summary of 3D QSAR pharmacophor model results

**r**^2^**Conventional**	**0.695**
Standard error of estimate	2.963
F Value	47.491
P Value	0.363
Significance Level	0.0500

#### Molecular alignment

**Figure 3:**
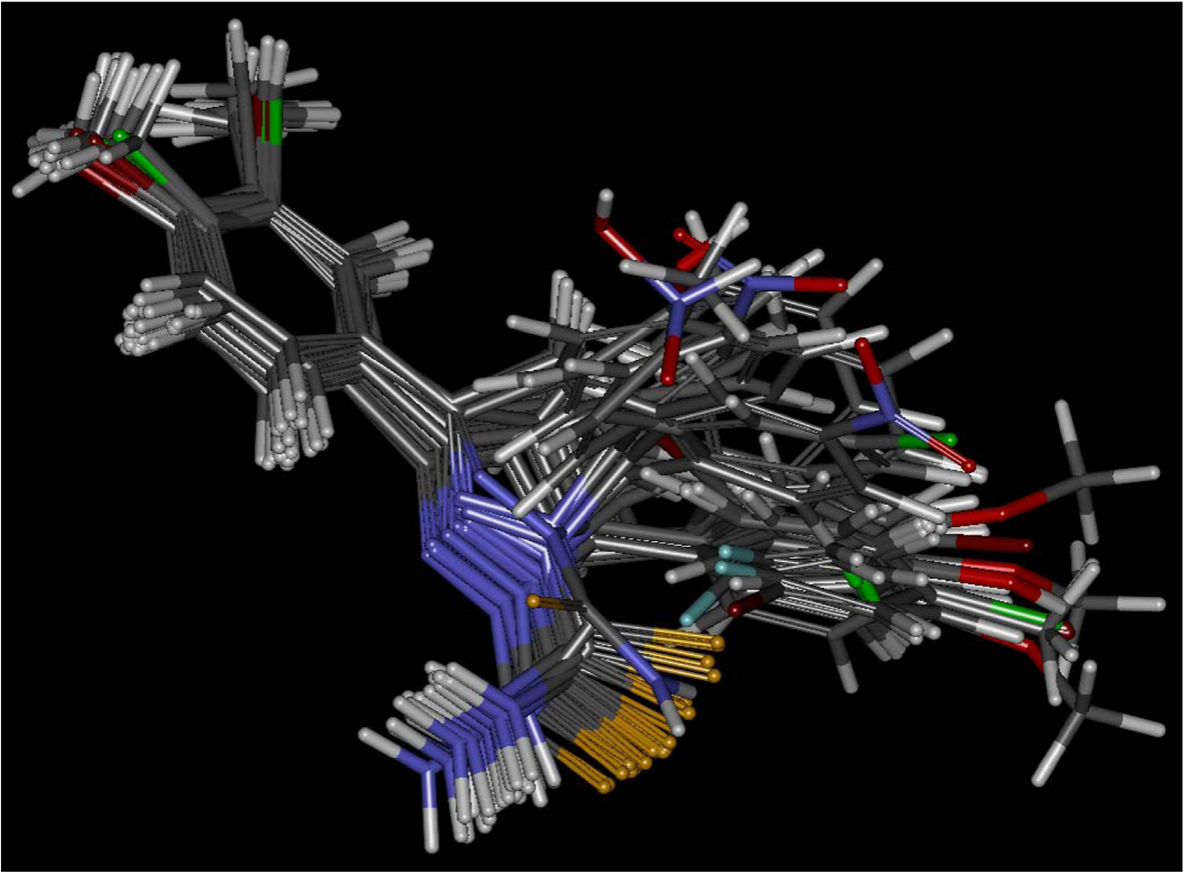
Alignment of compounds D1-30 with the best 3D QSAR pharmacophore.

However, for most studies, the reliability and efficiency of a CoMFA analysis depend on the correct molecular alignment of the ligands. So we performed a pharmacophore mapping study to select both a proposed bioactive conformer and a superposition rule [23]. It is naturally deduced that the pharmacophore model thus obtained provides a good starting point for CoMFA studies. In CATALYST, the studied molecules were automatically superimposed on the best pharmacophore model in generation hypothesis workbench using the generated conformers. For each ligand, one aligned conformer based on the lowest RMS deviation of feature atom coordinates from those of the corresponding reference features was superimposed on the hypothesis. In the studying process, we found that the best alignment based on the lowest RMS sometimes was not the most appropriate for a subsequent CoMFA analysis, and artificial adjustment was usually needed to get the reasonable alignment, as shown in Figure 3. In most cases of the CoMFA calculations, the alignment from the simple atom-by-atom fits may be the most widely used procedure. So a rigid alignment was applied to superimpose d-5 compounds onto an unsubstituented template shown in Figure 2 using an atom-by-atom least-squares fit as implemented in the SYBYL FIT option, and compound d-5 with the best biological activity was treated as the reference molecule. A conventional CoMFA was performed with the usually used steric and electrostatic fields implemented in SYBYL. The atomic Gasteiger charges were applied in the determination of the electrostatic field. All CoMFA calculations were performed with SYBYL standard setup (steric and electrostatic fields with Lennard-Jones and Coulomb-type potentials, dielectric constant 1/*r*, cutoff 30 kcal/mol) using an sp3 carbon atom with a charge of +1.0. The extent and the orientation of the grids surrounding the tested molecules were the same as the grid spacing was set to 2 Å. 

#### GRID size

Once the molecules are aligned a grid or lattice is established which surrounds the set of analogs in potential receptor space. Current CoMFA studies seldom use grid resolution less than 1 Å and, most often, 2 Å. The choice of grid resolution represents a compromise between computational practicality and detailing of the fields. If the grid resolution is too small, the number of field–points (cells) becomes too large to perform a timely analysis. Moreover spatial information on field preference can be lost, through a ‘smearing out’ effect, if the cells become too small. The grid resolution in the 1 Å to 2 Å range corresponds to, at best, differentiating single carbon-carbon (1.54 Å) from one another.

#### CoMFA interaction energy

The steric and electrostatic (potential fields) energies were calculated at each lattice intersection of a regularly spaced grid box. The lattice spacing was set a value of 2.0 Å. CoMFA region was defined automatically which extends the lattice walls beyond the dimensions of each structure by 4.0 Å in all directions. The Lennard-Jones potential and coulomb potential energy which represent, respectively steric and electrostatic fields, were calculated using the TRIPOS force fields. A sp3 carbon atom with a van der Waals radius of 1.52 Å and a +1.0 charge served as the probe atom to calculate steric and electrostatic fields. The default value 30.0 kcal/mole was used as the maximum electrostatic and steric energy cutoff.

#### Partial least squares (PLS) and cross-validation in CoMFA

**Figure 4:**
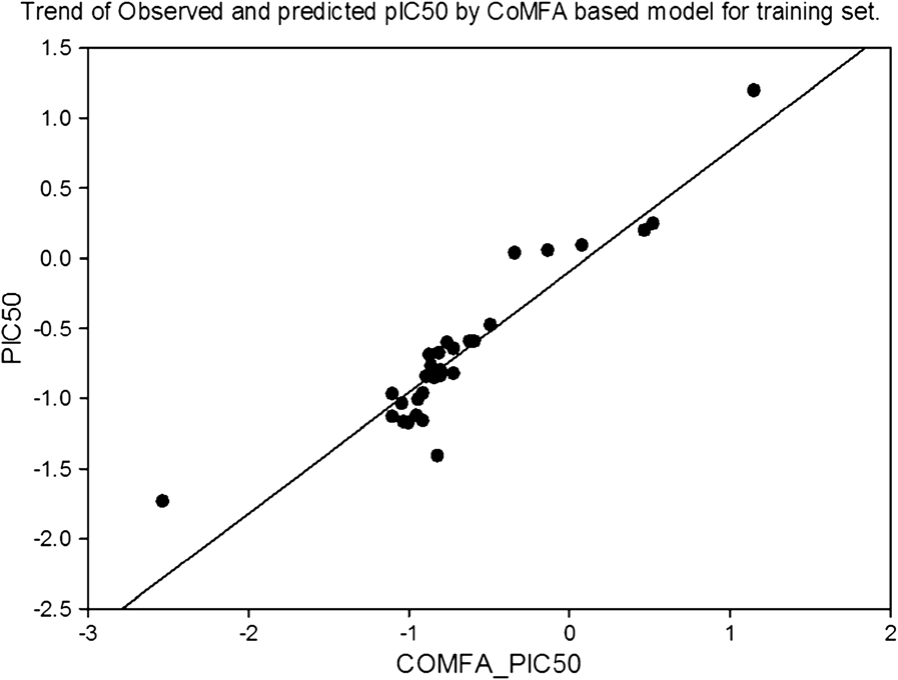
Correlation line of COMFA_PIC50 vs PIC50 of the pharmacophore model.

The last step in a CoMFA is a partial least square analysis to determine the minimal set of grid points which is necessary to explain the biological activities of the compounds. Partial least–square is an iterative procedure that applies two criteria to produce its solution. First, to extract a new component, the criterion is to maximize the degree of commonality between all of the structural parameter columns (independent variable) collectively and the experimental data (dependent variable). Second, in the evaluation phase of a PLS iteration, the criterion for acceptance of the principal component just generated is an improvement in the ability to predict, not to reproduce, the dependent variable, as outlined in Figure 4.

**Figure 5:**
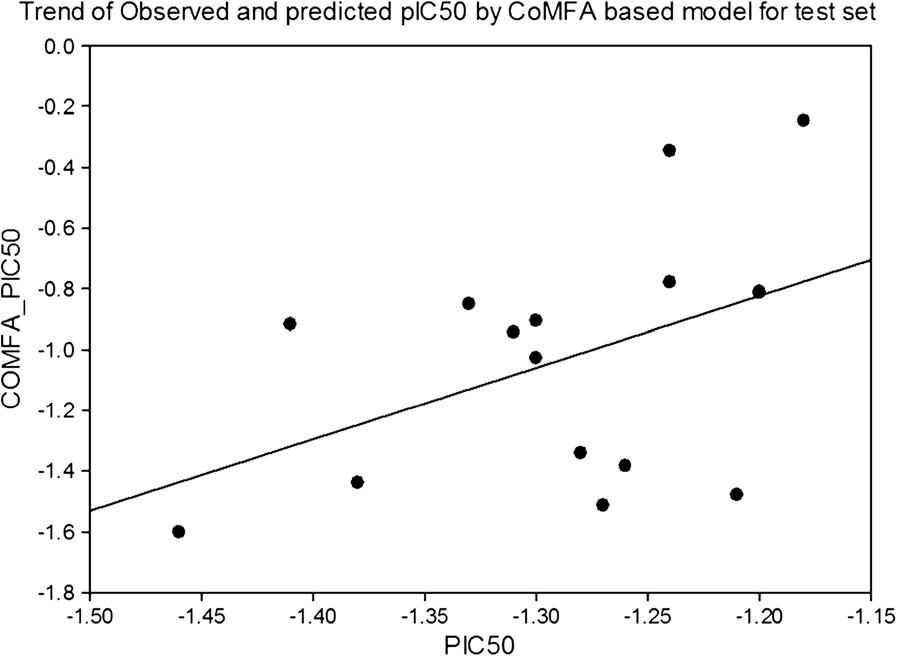
Correlation line of PIC50 vs COMFA_PIC50 of the pharmacophore model.

The technique used in PLS to assess the predictive ability of a QSAR is cross-validation [24]. Cross-validation is based on the idea that the best way to assess predictive performance is to predict. When cross-validating, one pretends that one or more of the unknown experimental value is, infect, unknown. The analysis being cross-validated is repeated, excluding the temporarily ‘unknown’ compounds and then using the resulting equation to predict the experimental measurement of the omitted compound(s). The cross-validation cycle is repeated until each compound has been excluded and predicted exactly once. The results of cross-validation are the sum of the squared prediction errors, sometimes called the predicted residual sum of squares (PRESS). For evaluation of the overall analysis, the PRESS is commonly expressed as a cross–validated correlation coefficient r^2^, or xv - r^2^ value, as outlined in Figure 5. 

The results of the CoMFA studies are summarized in Table 4. From this table it is evident that the CoMFA derived 3D QSAR shows a good cross validated r^2^, (0.568) and conventional r^2^, 0.895, therefore indicates a considerable predictive and correlative capacity of the model as shown in Table 5. In this analysis both steric and electrostatic field contribute to the 3D QSAR equation by 73.8% and 26.2%, respectively, suggesting that variation in biological activity of compounds is dominated by differences in steric (van der Waals) interactions.

**Table 4:** Summary of COMFA results*

**r**^2^**Conventional**	**0.895**
Standard error of estimate	0.163
F Value	(n1 = 6, n2 = 23) 24.478
P Value	0.000
r^2^ cross-validated	0.568
Standard error of predictions	0.21
No. of components	6
Steric contribution	0.454
Electrostatic contribution	0.546
Mean PIC_50_	−0.8896
Mean predicted PIC_50_	−0.799

^*^Results from leave one out (LOO) cross validation analysis using six components.

**Table 5:** Data from partial least squares (PLS) cross-validated analysis of training set

**Compounds**	**PIC**_**50**_	**COMFA**	**PRE_PIC**_**50**_	**Residual**
**D1**	0.0810	140.0000	0.089	−0.008
**D2**	−0.1340	142.0000	0.052	−0.186
**D3**	−0.3400	146.0000	0.035	−0.375
**D4**	0.4700	146.0000	0.194	0.276
**D5**	1.1500	146.0000	1.196	−0.046
**D6**	0.5230	140.0000	0.244	0.279
**D7**	−0.4900	156.0000	−0.476	−0.014
**D8**	−0.7200	134.0000	−0.828	0.108
**D9**	−0.5900	138.0000	−0.597	0.007
**D10**	−0.6200	138.0000	−0.597	−0.023
**D11**	−0.8000	134.0000	−0.805	0.005
**D12**	−0.8000	136.0000	−0.842	0.042
**D13**	−0.8400	140.0000	−0.856	0.016
**D14**	−0.8700	142.0000	−0.691	−0.179
**D15**	−0.7600	142.0000	−0.605	−0.155
**D16**	−0.7200	136.0000	−0.648	−0.072
**D17**	−0.8900	142.0000	−0.848	−0.042
**D18**	−0.8200	136.0000	−1.412	0.892
**D19**	−0.8600	136.0000	−0.771	−0.089
**D20**	−0.8100	138.0000	−0.679	−0.131
**D21**	−0.9500	136.0000	−1.127	0.177
**D22**	−0.9100	138.0000	−1.164	0.254
**D23**	−1.0000	142.0000	−1.178	0.178
**D24**	−0.9400	144.0000	−1.013	0.073
**D25**	−0.9100	144.0000	−0.968	0.058
**D26**	−1.1000	138.0000	−0.972	0.128
**D27**	−1.0300	144.0000	−1.170	0.14
**D28**	−2.5300	138.0000	−1.735	−0.795
**D29**	−1.135	138.0000	−1.1000	−0.035
**D30**	−1.042	140.0000	−1.0400	−0.002

The real test for model predictiveness is to predict the activity of ligands, which were not used in the model generation. Our test set has 16 compounds, which were randomly kept with bias given to both chemical and biological diversity in both the training set and the test set molecules, as shown in Tables 6.

**Table 6:** Data from PLS cross-validated analysis of test set

**Compounds**	**PIC**_**50**_	**COMFA**	**PRE_PIC**_**50**_	**Residual**
**F1**	−1.2600	134.0000	−1.384	−0.124
**F2**	−1.2100	134.0000	−1.480	−0.27
**F3**	−1.2700	138.0000	−1.514	−0.244
**F4**	−1.2800	144.0000	−1.342	−0.062
**F5**	−1.2000	146.0000	−0.814	0.386
**F6**	−1.3800	134.0000	−1.440	−0.06
**F7**	−1.4600	132.0000	−1.602	−0.142
**F8**	−1.4100	156.0000	−0.918	0.492
**F9**	−1.2000	136.0000	−0.811	0.389
**F10**	−1.3000	136.0000	−0.906	0.494
**F11**	−1.3100	140.0000	−0.944	0.366
**F12**	−1.2400	146.0000	−0.780	0.46
**F13**	−1.1800	148.0000	−0.247	0.933
**F14**	−1.3300	136.0000	−0.851	0.479
**F15**	−1.3000	134.0000	−1.028	0.272
**F16**	−1.2400	158.0000	−0.346	0.894

The CoMFA models exhibited a good predictiveness on these ligands with conventional r^2^ 0.469 and standard error of estimation 0.372 as the data described in Table 7. The contour maps are plotted as percentage contribution to the QSAR equation and are associated with the differences in biological activity.

**Table 7:** Data from partial least squares (PLS) cross-validated analysis of test set

**r**^2^**Conventional**	**0.469**
Standard error of estimate	0.372
P Value	0.0713
r^2^ cross-validated	0.268
No. of components	6
Steric contribution	0.454
Electrostatic contribution	0.546
Mean PIC_50_	−1.2856
Mean predicted PIC_50_	−1.0254

^*^Results from leave one out (LOO) cross validation analysis using six components.

**Figure 6:**
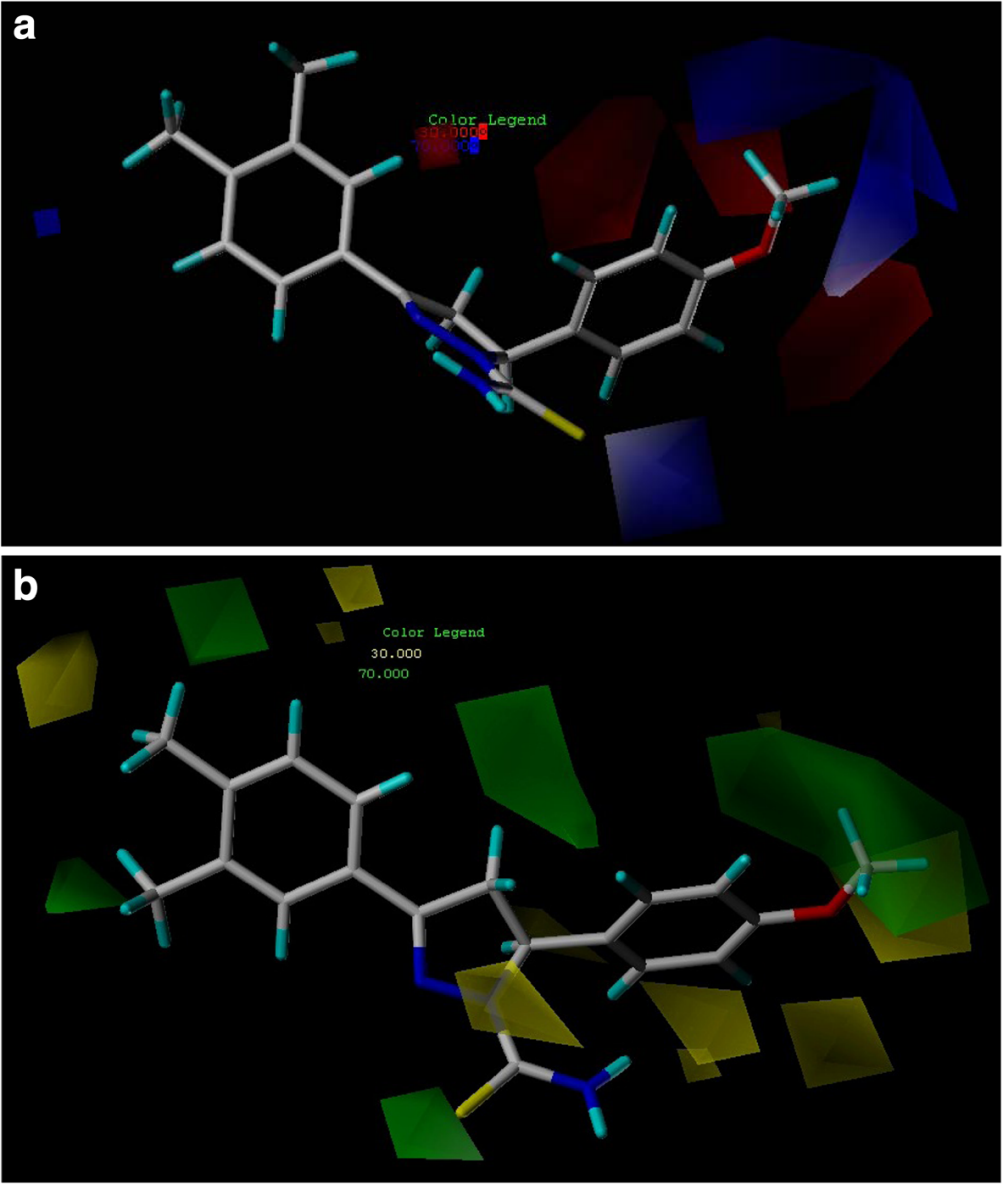
(**a). Electrostatic contour plot: positive (contribution level of 70%) and negative (contribution level of 30%) charge favoring areas are represented as blue and red contours, respectively (b).** Steric contour plot: favored (contribution level 70%) and unflavored (contribution level 30%) areas are represented as green and yellow contours, respectively.

In Figures 6(a) the regions of high and low steric tolerance are shown in green and yellow polyhedral, respectively. The areas of high bulk tolerance (80% contribution) are observed near 4-methoxy phenyl of the ligands . The active analogue (5) outlined in Figure 6(b), shows that 4-methoxy phenyl ring embedded in the green region. The antitumor activity shown by the different training compounds was due to the presence of bulky groups at position 5 and 3, 4-disbstituted phenyl at position 3 surrounded by green contours. In the present sterically unflavored yellow regions were observed near the 3, 6-substituted phenyl at position 3. The steric bulk in this region has a negative effect on the antitumor activity, suggesting that there is a definite requirement of a substructure with appropriate shape to exhibit high activity.

#### CoMFA applications in drug design

There are now a few hundred practical applications of CoMFA in drug design. Most applications are in the field of ligand protein interactions, describing affinity or inhibition constants. In addition, CoMFA has been used to correlate steric and electronic parameters [25]. Less appropriate seems the application of CoMFA to in vivo data, even if lipophilicity is considered as an additional parameter. As most CoMFA applications in drug design have been comprehensively reviewed in three books [26,27] and in some reviews [28,29].

## Conclusion

This work demonstrated that the 3D QSAR pharmacophore modeling and MFA technique (CoMFA) could be effectively combined. The best model identified by 3d QSAR pharmacophore search served as suitable modes of superimposition for subsequent CoMFA utilizing compounds (D1-30), as training set. The better results were obtained by superimposing the molecules onto the 3D QSAR pharmacophore model than onto the common structure used in the energy-lowest conformers. Moreover, from our calculations, it can be found that the active conformers and the 3D QSAR pharmacophore mapping automatically afforded by CATALYST are usually not the optimal ones. The manual adjustments are usually needed to obtain the best results. As illustrated by the contour maps, our 3D-QSAR model, which is based on a homogeneous set of ligands, is expected to correctly predict affinities of structurally related compounds. These results will provide useful information in understanding the structural and chemical features required for EAC cells cytotoxicity and in designing new potential compounds.

## Experimental

### Chemistry

Separation of the compounds by column chromatography was carried out with silica gel 60 (200–300 mesh ASTM, E. Merck). The quantity of silica gel used was 50–100 times the weight charged on the column. Then, the eluates were monitored using TLC. Melting points (uncorrected) were determined on a XT4 MP apparatus (Ain Shams University, Egypt). Mass spectra were recorded on a Varian MAT 112 spectrometer. Analytical data were obtained from the Microanalytical Data Center at Cairo University and some compounds at Ain Shams University. The ^1^ H NMR spectra were recorded on a Varian 400 MHz spectrometer at 25°C with TMS and solvent signals allotted as internal standards. Chemical shifts were reported in ppm (*δ*). Elemental analyses were performed on a CHN-O-Rapid instrument and were within ±0.4% of the theoretical values obtained from the Microanalytical Data Center at Cairo University.

#### General synthetic procedure of chalcones

Equimolar portions of the appropriately substituted aromatic aldehydes (10 mmol, 1 equiv) and ketones (10 mmol, 1 equiv) were dissolved in approximately 15 mL of ethanol. The mixture was allowed to stir for several minutes at 5–10°C. A 10 mL aliquot of a 40% aqueous sodium hydroxide solution was then slowly added dropwise to the reaction flask via a self-equalizing addition funnel. The reaction solution was allowed to stir at room temperature for approximately 4 h. Most commonly, a precipitate formed and was then collected by suction filtration.

#### General synthetic procedure of pyrazole derivatives C1–C30

A mixture of chalcone (0.01 mol), thiosemicarbazide (0.01 mol), and NaOH (0.025 mol) was refluxed in ethanol (25 mL) for 8 h. The solution was poured into ice-water. The precipitate was filtered and crystallized from methanol to give white powder compounds in good to excellent yields.

#### 3-(3,4-Dimethylphenyl)-5-(4-fluorophenyl)-4,5-dihydro-1 H-pyrazole-1-carbothioamide (D1)

Appearance: white powder**.** Melting Point**:** 223–225°C. ^1^ H NMR (300 MHz, DMSO-d6): 2.29 (s, 6 H); 3.12– 3.18 (dd, J1 = 3.66, J2 = 17.58 Hz, 1Ha); 3.74–3.83 (dd, J1 = 11.52, J2 = 17.76 Hz, 1Hc); 5.92–5.97 (dd, J1 = 3.66, J2 = 11.56 Hz, 1Hb); 7.09–7.18 (m, 3 H); 7.42–7.46 (m, 2 H); 7.46–7.48 (m, 1 H); 7.50 (s, 1 H). MS (ESI): 328.1 (C18H19FN3S, [M + H]^+^). Anal. Calcd for C18H18FN3S: C, 66.03; H, 5.54; N, 12.83. Found: C, 66.17; H, 5.72; N, 12.65.

#### 5-(4-Chlorophenyl)-3-(3,4-dimethylphenyl)-4,5-dihydro-1 H-pyrazole-1-carbothioamide (D2)

Appearance: white powder**.** Melting Point**:** 216–217°C. ^1^ H NMR (300 MHz, DMSO-d6): 2.30 (s, 6 H); 3.11– 3.18 (dd, J1 = 3.66, J2 = 17.73 Hz, 1Ha); 3.76–3.86 (dd, J1 = 11.52, J2 = 17.73 Hz, 1Hc); 5.96–6.01 (dd, J1 = 3.66, J2 = 11.34 Hz, 1Hb); 7.14–7.19 (m, 3 H); 7.27–7.30 (m, 2 H); 7.41–7.44 (m, 1 H); 7.50 (s, 1 H). MS (ESI): 344.1 (C18H19ClN3S, [M + H]^+^). Anal. Calcd for C18H18ClN3S: C, 62.87; H, 5.28; N, 12.22. Found: C, 62.69; H, 5.42; N, 12.41.

#### 5-(4-Bromophenyl)-3-(3,4-dimethylphenyl)-4,5-dihydro-1 H-pyrazole-1-carbothioamide (D3)

Appearance: white powder**.** Melting Point**:** 231–233°C. ^1^ H NMR (300 MHz, DMSO-d6): 2.30 (s, 6 H); 3.11– 3.18 (dd, J1 = 3.66, J2 = 17.55 Hz, 1Ha); 3.76–3.86 (dd, J1 = 11.52, J2 = 17.76 Hz, 1Hc); 5.94–5.99 (dd, J1 = 3.66, J2 = 11.52 Hz, 1Hb); 7.08–7.19 (m, 3 H); 7.41–7.44 (m, 2 H); 7.45–7.47 (m, 1 H); 7.51 (s, 1 H). MS (ESI): 388.1 (C18H19BrN3S, [M + H]^+^). Anal. Calcd for C18H18BrN3S: C, 55.67; H, 4.67; N, 10.82. Found: C, 55.78; H, 4.81; N, 10.64.

#### 3-(3,4-Dimethylphenyl)-5-p-tolyl-4,5-dihydro-1 H-pyrazole-1-carbothioamide (D4)

Appearance: white powder**.** Melting Point**:** 198–200°C. ^1^ H NMR (300 MHz, DMSO-d6): 2.30 (s, 9 H); 3.14–3.21 (dd, J1 = 3.48, J2 = 17.55 Hz, 1 H); 3.74–3.84 (dd, J1 = 11.34, J2 = 17.55 Hz, 1 H); 5.96–6.01 (dd, J1 = 3.45, J2 = 11.34 Hz, 1 H); 7.08–7.13 (m, 3 H); 7.14–7.18 (m, 2 H); 7.41–7.44 (m, 1 H); 7.51 (s, 1 H). MS (ESI): 324.1 (C19H22N3S, [M + H]^+^). Anal. Calcd for C19H21N3S: C, 70.55; H, 6.54; N, 12.99. Found: C, 70.74; H, 6.42; N, 12.76.

#### 3-(3,4-Dimethylphenyl)-5-(4-methoxyphenyl)-4,5-dihydro-1 H-pyrazole-1-carbothioamide (D5)

Appearance: white powder**.** Melting Point**:** 207–209°C. ^1^ H NMR (300 MHz, DMSO-d6): 2.31 (s, 6 H); 3.11– 3.19 (dd, J1 = 3.47, J2 = 17.56 Hz, 1 H); 3.71 (s, 3 H); 3.72–3.81 (dd, J1 = 11.42, J2 = 17.55 Hz, 1 H); 5.97–6.03 (dd, J1 = 3.45, J2 = 11.34 Hz, 1 H); 7.06–7.11 (m, 3 H); 7.15–7.18 (m, 2 H); 7.42–7.45 (m, 1 H); 7.51 (s, 1 H). MS (ESI): 340.1 (C19H22N3OS, [M + H]^+^). Anal. Calcd for C19H21N3OS: C, 67.23; H, 6.24; N, 12.38. Found: C, 67.48; H, 6.46; N, 12.17.

#### 3-(3,4-Dimethylphenyl)-5-(4-hydroxyphenyl)-4,5-dihydro-1 H-pyrazole-1-carbothioamide (D6)

Appearance: white powder**.** Melting Point**:** 218–219°C. ^1^ H NMR (300 MHz, DMSO-d6): 2.30 (s, 6 H); 3.12– 3.18 (dd, J1 = 3.67, J2 = 17.55 Hz, 1 H); 3.74–3.83 (dd, J1 = 11.53, J2 = 17.76 Hz, 1 H); 5.93–5.99 (dd, J1 = 3.68, J2 = 11.53 Hz, 1 H); 7.09–7.19 (m, 3 H); 7.41–7.44 (m, 2 H); 7.44–7.46 (m, 1 H); 7.50 (s, 1 H). MS (ESI): 326.1 (C18H20N3OS, [M + H]^+^). Anal. Calcd for C18H19N3OS: C, 66.43; H, 5.88; N, 12.91. Found: C, 66.65; H, 5.69; N, 12.75.

#### 3-(3,4-Dimethylphenyl)-5-(4-nitrophenyl)-4,5-dihydro1H-pyrazole-1-carbothioamide(D7)

Appearance: white powder**.** Melting Point**:** 202– 203°C. ^1^ H NMR (300 MHz, DMSO-d6): 2.31 (s, 6 H); 3.13–3.19 (dd, J1 = 3.66, J2 = 17.55 Hz, 1 H); 3.76–3.85 (dd, J1 = 11.51, J2 = 17.73 Hz, 1 H); 5.92–5.97 (dd, J1 = 3.65, J2 = 11.51 Hz, 1 H); 7.07–7.16 (m, 3 H); 7.40–7.43 (m, 2 H); 7.45–7.47 (m, 1 H); 7.50 (s, 1 H). MS (ESI): 355.1 (C18H19N4O2S, [M + H]^+^). Anal. Calcd for C18H18N4O2S: C, 61.00; H, 5.12; N, 15.81. Found: C, 61.18; H, 5.32; N, 15.64.

#### 3-(3,4-Dimethylphenyl)-5-(2-fluorophenyl)-4,5-dihydro-1 H-pyrazole-1-carbothioamide (D8)

Appearance: white powder**.** Melting Point**:** 211–213°C. ^1^ H NMR (300 MHz, DMSO-d6): 2.30 (s, 6 H); 3.08– 3.15 (dd, J1 = 4.04, J2 = 17.89 Hz, 1 H); 3.88–3.97 (dd, J1 = 11.53, J2 = 17.96 Hz, 1 H); 6.33–6.38 (dd, J1 = 4.09, J2 = 11.43 Hz, 1 H); 7.08–7.12 (m, 1 H); 7.16–7.26 (m, 3 H); 7.39–7.45 (m, 2 H); 7.51 (s, 1 H). MS (ESI): 328.1 (C18H19FN3S, [M + H]^+^). Anal. Calcd for C18H18FN3S: C, 66.03; H, 5.54; N, 12.83. Found: C, 66.19; H, 5.31; N, 12.74.

#### 5-(2-Chlorophenyl)-3-(3,4-dimethylphenyl)-4,5-dihydro-1 H-pyrazole-1-carbothioamide (D9)

Appearance: white powder**.** Melting Point**:** 208–210°C. ^1^ H NMR (300 MHz, DMSO-d6): 2.29 (s, 6 H); 3.06–3.14 (dd, J1 = 4.02, J2 = 17.94 Hz, 1 H); 3.85–3.95 (dd, J1 = 11.52, J2 = 17.91 Hz, 1 H); 6.30–6.35 (dd, J1 = 4.05, J2 = 11.52 Hz, 1 H); 7.04–7.07 (m, 1 H); 7.15–7.23 (m, 3 H); 7.37–7.44 (m, 2 H); 7.50 (s, 1 H). MS (ESI): 344.1 (C18H19ClN3S, [M + H]^+^). Anal. Calcd for C18H18ClN3S: C, 62.87; H, 5.28; N, 12.22. Found: C, 62.63; H, 5.38; N, 12.46.

#### 5-(2-Bromophenyl)-3-(3,4-dimethylphenyl)-4,5-dihydro-1 H-pyrazole-1-carbothioamide (D10)

Appearance: white powder**.** Melting Point**:** 217–219°C. ^1^ H NMR (300 MHz, DMSO-d6): 2.31 (s, 6 H); 3.05–3.12 (dd, J1 = 4.06, J2 = 17.96 Hz, 1 H); 3.83–3.94 (dd, J1 = 11.51, J2 = 17.93 Hz, 1 H); 6.28–6.32 (dd, J1 = 4.03, J2 = 11.52 Hz, 1 H); 7.06– 7.09 (m, 1 H); 7.16–7.23 (m, 3 H); 7.38–7.45 (m, 2 H); 7.50 (s, 1 H). MS (ESI): 388.1 (C18H19BrN3S, [M + H]^+^). Anal. Calcd for C18H18BrN3S: C, 55.67; H, 4.67; N, 10.82. Found: C, 55.83; H, 4.54; N, 10.74.

#### 3-(3,4-Dichlorophenyl)-5-(4-fluorophenyl)-4,5-dihydro-1 H-pyrazole-1-carbothioamide (D11)

Appearance: white powder**.** Melting Point**:** 206–208°C. ^1^ H NMR (300 MHz, DMSO-d6): 3.11–3.17 (dd, J1 = 3.65, J2 = 17.51 Hz, 1 H); 3.72–3.83 (dd, J1 = 11.32, J2 = 17.73 Hz, 1 H); 5.91–5.96 (dd, J1 = 3.67, J2 = 11.51 Hz, 1 H); 7.07–7.16 (m, 3 H); 7.41–7.45 (m, 2 H); 7.47–7.48 (m, 1 H); 7.50 (s, 1 H). MS (ESI): 368.0 (C16H13Cl2FN3S, [M + H]^+^). Anal. Calcd for C16H12Cl2FN3S: C, 52.18; H, 3.28; N, 11.41. Found: C, 52.35; H, 3.41; N, 11.34.

#### 5-(4-Chlorophenyl)-3-(3,4-dichlorophenyl)-4,5-dihydro-1 H-pyrazole-1-carbothioamide (D12)

Appearance: white powder**.** Melting Point**:** 211–213°C. ^1^ H NMR (300 MHz, DMSO-d6): 3.11–3.17 (dd, J1 = 3.68, J2 = 17.75 Hz, 1 H); 3.75–3.86 (dd, J1 = 11.51, J2 = 17.73 Hz, 1 H); 5.94–6.02 (dd, J1 = 3.65, J2 = 11.37 Hz, 1 H); 7.13–7.17 (m, 3 H); 7.28–7.32 (m, 2 H); 7.40–7.44 (m, 1 H); 7.51 (s, 1 H). MS (ESI): 383.0 (C16H13Cl3N3S, [M + H]^+^). Anal. Calcd for C16H12Cl3N3S: C, 49.95; H, 3.14; N, 10.92. Found: C, 49.82; H, 3.26; N, 10.78.

#### 5-(4-Bromophenyl)-3-(3,4-dichlorophenyl)-4,5-dihydro-1 H-pyrazole-1-carbothioamide (D13)

Appearance: white powder**.** Melting Point**:** 214–216°C. ^1^ H NMR (300 MHz, DMSO-d6): 3.12–3.18 (dd, J1 = 3.68, J2 = 17.53 Hz, 1 H); 3.74–3.86 (dd, J1 = 11.53, J2 = 17.76 Hz, 1 H); 5.92–5.98 (dd, J1 = 3.66, J2 = 11.53 Hz, 1 H); 7.07–7.17 (m, 3 H); 7.42–7.45 (m, 2 H); 7.47–7.48 (m, 1 H); 7.51 (s, 1 H). MS (ESI): 427.0 (C16H13BrCl2N3S, [M + H]^+^). Anal. Calcd for C16H12BrCl2N3S: C, 44.78; H, 2.82; N, 9.79. Found: C, 44.96; H, 2.66; N, 9.98.

#### 3-(3,4-Dichlorophenyl)-5-p-tolyl-4,5-dihydro-1 H-pyrazole-1-carbothioamide (D14)

Appearance: white powder**.** Melting Point**:** 223–225°C. ^1^ H NMR (300 MHz, DMSO-d6): 2.30 (s, 3 H); 3.12–3.21 (dd, J1 = 3.46, J2 = 17.55 Hz, 1 H); 3.76–3.84 (dd, J1 = 11.37, J2 = 17.53 Hz, 1 H); 5.94–6.01 (dd, J1 = 3.46, J2 = 11.34 Hz, 1 H); 7.06–7.13 (m, 3 H); 7.13–7.17 (m, 2 H); 7.41–7.43 (m, 1 H); 7.50 (s, 1 H). MS (ESI): 364.0 (C17H16Cl2N3S, [M + H]^+^). Anal. Calcd for C17H15Cl2N3S: C, 56.05; H, 4.15; N, 11.53. Found: C, 56.18; H, 4.26; N, 11.38.

#### 3-(3,4-Dichlorophenyl)-5-(4-methoxyphenyl)-4,5-dihydro-1 H-pyrazole-1-carbothioamide (D15)

Appearance: white powder**.** Melting Point**:** 209–210°C. ^1^ H NMR (300 MHz, DMSO-d6): 3.12–3.19 (dd, J1 = 3.46, J2 = 17.53 Hz, 1 H); 3.71 (s, 3 H); 3.74–3.81 (dd, J1 = 11.43, J2 = 17.53 Hz, 1 H); 5.94–6.01 (dd, J1 = 3.46, J2 = 11.34 Hz, 1 H); 7.07–7.11 (m, 3 H); 7.14–7.18 (m, 2 H); 7.42–7.45 (m, 1 H); 7.51 (s, 1 H). MS (ESI): 380.0 (C17H16Cl2N3OS, [M + H]^+^). Anal. Calcd for C17H15Cl2N3OS: C, 53.69; H, 3.98; N, 11.05. Found: C, 53.78; H, 3.81; N, 11.24.

#### 3-(3,4-Dichlorophenyl)-5-(4-hydroxyphenyl)-4,5-dihydro-1 H-pyrazole-1-carbothioamide (D16)

Appearance: white powder**.** Melting Point**:** p 235–237°C. ^1^ H NMR (300 MHz, DMSO-d6): 3.12–3.17 (dd, J1 = 3.67, J2 = 17.53 Hz, 1 H); 3.72–3.83 (dd, J1 = 11.56, J2 = 17.76 Hz, 1 H); 5.92–5.98 (dd, J1 = 3.68, J2 = 11.51 Hz, 1 H); 7.08–7.19 (m, 3 H); 7.42–7.44 (m, 2 H); 7.44–7.47 (m, 1 H); 7.50 (s, 1 H). MS (ESI): 366.0 (C16H14Cl2N3OS, [M + H]^+^). Anal. Calcd for C16H13Cl2N3OS: C, 52.47; H, 3.58; N, 11.47. Found: C, 52.62; H, 3.74; N, 11.59.

#### 3-(3,4-Dichlorophenyl)-5-(4-nitrophenyl)-4,5-dihydro1H-pyrazole-1-carbothioamide (D17)

Appearance: white powder**.** Melting Point**:** 203– 205°C. ^1^ H NMR (300 MHz, DMSO-d6): 3.13–3.18 (dd, J1 = 3.65, J2 = 17.53 Hz, 1 H); 3.74–3.83 (dd, J1 = 11.53, J2 = 17.73 Hz, 1 H); 5.94–5.97 (dd, J1 = 3.65, J2 = 11.53 Hz, 1 H); 7.07–7.14 (m, 3 H); 7.40–7.43 (m, 2 H); 7.46–7.48 (m, 1 H); 7.50 (s, 1 H). MS (ESI): 395.0 (C16H13Cl2N4O2S, [M + H]^+^). Anal. Calcd for C16H12Cl2N4O2S: C, 48.62; H, 3.06; N, 14.17. Found: C, 48.76; H, 3.24; N, 14.34.

#### 3-(3,4-Dichlorophenyl)-5-(2-fluorophenyl)-4,5-dihydro-1 H-pyrazole-1-carbothioamide (D18)

Appearance: white powder**.** Melting Point**:** 235–236°C. ^1^ H NMR (300 MHz, DMSO-d6): 3.09–3.15 (dd, J1 = 4.16, J2 = 17.86 Hz, 1 H); 3.85–3.96 (dd, J1 = 11.53, J2 = 17.96 Hz, 1 H); 6.31–6.38 (dd, J1 = 4.21, J2 = 11.43 Hz, 1 H); 7.08–7.14 (m, 1 H); 7.18–7.26 (m, 3 H); 7.36–7.43 (m, 2 H); 7.51 (s, 1 H). MS (ESI): 368.0 (C16H13Cl2FN3S, [M + H]^+^). Anal. Calcd for C16H12Cl2FN3S: C, 52.18; H, 3.28; N, 11.41. Found: C, 52.32; H, 3.39; N, 11.58.

#### 5-(2-Chlorophenyl)-3-(3,4-dichlorophenyl)-4,5-dihydro-1H-pyrazole-1-carbothioamide (D19)

Appearance: white powder**.** Melting Point**:** 241–243°C. ^1^ H NMR (300 MHz, DMSO-d6): 3.06–3.12 (dd, J1 = 4.14, J2 = 17.92 Hz, 1 H); 3.87–3.95 (dd, J1 = 11.53, J2 = 17.91 Hz, 1 H); 6.31–6.35 (dd, J1 = 4.26, J2 = 11.52 Hz, 1 H); 7.03–7.07 (m, 1 H); 7.17–7.23 (m, 3 H); 7.36–7.44 (m, 2 H); 7.50 (s, 1 H). MS (ESI): 383.0 (C16H13Cl3N3S, [M + H]^+^). Anal. Calcd for C16H12Cl3N3S: C, 49.95; H, 3.14; N, 10.92. Found: C, 49.76; H, 3.27; N, 10.81.

#### 5-(2-Bromophenyl)-3-(3,4-dichlorophenyl)-4,5-dihydro-1 H-pyrazole-1-carbothioamide (D20)

Appearance: white powder**.** Melting Point**:** 229–230°C. ^1^ H NMR (300 MHz, DMSO-d6): 3.07–3.12 (dd, J1 = 4.16, J2 = 17.93 Hz, 1 H); 3.86–3.94 (dd, J1 = 11.53, J2 = 17.91 Hz, 1 H); 6.26–6.32 (dd, J1 = 4.13, J2 = 11.51 Hz, 1 H); 7.04–7.09 (m, 1 H); 7.17–7.23 (m, 3 H); 7.36–7.45 (m, 2 H); 7.50 (s, 1 H). MS (ESI): 427.0 (C16H13BrCl2N3S, [M + H]^+^). Anal. Calcd for C16H12BrCl2N3S: C, 44.78; H, 2.82; N, 9.79. Found: C, 44.92; H, 2.69; N, 9.93.

#### 3*-(3,4-Dibromophenyl)-5-(4-fluorophenyl)-4,5-dihydro-1 H-pyrazole-1-carbothioamide (D21)*

Appearance: white powder**.** Melting Point**:** 246–247°C. ^1^ H NMR (300 MHz, DMSO-d6): 3.14–3.17 (dd, J1 = 3.66, J2 = 17.51 Hz, 1 H); 3.76–3.83 (dd, J1 = 11.31, J2 = 17.73 Hz, 1 H); 5.92–5.96 (dd, J1 = 3.66, J2 = 11.56 Hz, 1 H); 7.05–7.16 (m, 3 H); 7.42–7.45 (m, 2 H); 7.46–7.48 (m, 1 H); 7.51 (s, 1 H). MS (ESI): 455.0 (C16H13Br2FN3S, [M + H]^+^). Anal. Calcd for C16H12Br2FN3S: C, 42.04; H, 2.65; N, 9.19. Found: C, 42.17; H, 2.74; N, 9.35.

#### 5-(4-Chlorophenyl)-3-(3,4-dibromophenyl)-4,5-dihydro-1 H-pyrazole-1-carbothioamide (D22)

Appearance: white powder**.** Melting Point**:** 216–218°C. ^1^ H NMR (300 MHz, DMSO-d6): 3.12–3.17 (dd, J1 = 3.66, J2 = 17.75 Hz, 1 H); 3.73–3.86 (dd, J1 = 11.53, J2 = 17.71 Hz, 1 H); 5.96–6.04 (dd, J1 = 3.65, J2 = 11.45 Hz, 1 H); 7.15–7.17 (m, 3 H); 7.26–7.32 (m, 2 H); 7.41–7.44 (m, 1 H); 7.51 (s, 1 H). MS (ESI): 471.0 (C16H13Br2ClN3S, [M + H]^+^). Anal. Calcd for C16H12Br2ClN3S: C, 40.58; H, 2.55; N, 8.87. Found: C, 40.46; H, 2.68; N, 8.69.

#### 5-(4-Bromophenyl)-3-(3,4-dibromophenyl)-4,5-dihydro-1 H-pyrazole-1-carbothioamide (D23)

Appearance: white powder**.** Melting Point**:** 239–241°C. ^1^ H NMR (300 MHz, DMSO-d6): 3.14–3.18 (dd, J1 = 3.69, J2 = 17.53 Hz, 1 H); 3.77–3.86 (dd, J1 = 11.51, J2 = 17.76 Hz, 1 H); 5.91–5.98 (dd, J1 = 3.66, J2 = 11.57 Hz, 1 H); 7.09–7.17 (m, 3 H); 7.42–7.46 (m, 2 H); 7.47–7.48 (m, 1 H); 7.50 (s, 1 H). MS (ESI): 515.0(C16H13Br3N3S, [M + H]^+^). Anal. Calcd for C16H12Br3N3S: C, 37.09; H, 2.33; N, 8.11. Found: C, 37.18; H, 2.45; N, 8.32.

#### 3-(3,4-Dibromophenyl)-5-p-tolyl-4,5-dihydro-1 H-pyrazole-1-carbothioamide (D24)

Appearance: white powder**.** Melting Point**:** 211–213°C. ^1^ H NMR (300 MHz, DMSO-d6): 2.30 (s, 3 H); 3.13–3.21 (dd, J1 = 3.46, J2 = 17.55 Hz, 1 H); 3.74–3.84 (dd, J1 = 11.31, J2 = 17.57 Hz, 1 H); 5.96–6.01 (dd, J1 = 3.46, J2 = 11.37 Hz, 1 H); 7.08–7.13 (m, 3 H); 7.13–7.17 (m, 2 H); 7.41–7.43 (m, 1 H); 7.50 (s, 1 H). MS (ESI): 451.0 (C17H16Br2N3S, [M + H]^+^). Anal. Calcd for C17H15Br2N3S: C, 45.05; H, 3.34; N, 9.27. Found: C, 45.21; H, 3.48; N, 9.36.

#### 3-(3,4-Dibromophenyl)-5-(4-methoxyphenyl)-4,5-dihydro-1 H-pyrazole-1-carbothioamide (D25)

Appearance: white powder**.** Melting Point**:** 236–238°C. ^1^ H NMR (300 MHz, DMSO-d6): 3.11–3.17 (dd, J1 = 3.43, J2 = 17.53 Hz, 1 H); 3.71 (s, 3 H); 3.77–3.81 (dd, J1 = 11.46, J2 = 17.53 Hz, 1 H); 5.97–6.01 (dd, J1 = 3.43, J2 = 11.34 Hz, 1 H); 7.08– 7.11 (m, 3 H); 7.16–7.18 (m, 2 H); 7.42–7.45 (m, 1 H); 7.51 (s, 1 H). MS (ESI): 467.0 (C17H16Br2N3OS, [M + H]^+^). Anal. Calcd for C17H15Br2N3OS: C, 43.52; H, 3.22; N, 8.96. Found: C, 43.68; H, 3.37; N, 8.84.

#### 3-(3,4-Dibromophenyl)-5-(4-hydroxyphenyl)-4,5-dihydro-1 H-pyrazole-1-carbothioamide (D26)

Appearance: white powder**.** Melting Point**:** 221–223°C. ^1^ H NMR (300 MHz, DMSO-d6): 3.11–3.16 (dd, J1 = 3.63, J2 = 17.51 Hz, 1 H); 3.75–3.83 (dd, J1 = 11.53, J2 = 17.76 Hz, 1 H); 5.93–5.98 (dd, J1 = 3.68, J2 = 11.53 Hz, 1 H); 7.09–7.19 (m, 3 H); 7.42–7.45 (m, 2 H); 7.46–7.48 (m, 1 H); 7.50 (s, 1 H). MS (ESI): 453.0 (C16H14Br2N3OS, [M + H]^+^). Anal. Calcd for C16H13Br2N3OS: C, 42.22; H, 2.88; N, 9.23. Found: C, 42.45; H, 2.69; N, 9.38.

#### 3-(3,4-Dibromophenyl)-5-(4-nitrophenyl)-4,5-dihydro1H-pyrazole-1-carbothioamide (D27)

Appearance: white powder**.** Melting Point**:** 246– 248°C. ^1^ H NMR (300 MHz, DMSO-d6): 3.12–3.18 (dd, J1 = 3.69, J2 = 17.53 Hz, 1 H); 3.76–3.83 (dd, J1 = 11.51, J2 = 17.73 Hz, 1 H); 5.92–5.97 (dd, J1 = 3.63, J2 = 11.53 Hz, 1 H); 7.09–7.14 (m, 3 H); 7.41–7.43 (m, 2 H); 7.46–7.48 (m, 1 H); 7.50 (s, 1 H). MS (ESI): 482.0 (C16H13Br2N4O2S, [M + H]^+^). Anal. Calcd for C16H12Br2N4O2S: C, 39.69; H, 2.50; N, 11.57. Found: C, 39.78; H, 2.41; N, 11.69.

#### 3-(3,4-Dibromophenyl)-5-(2-fluorophenyl)-4,5-dihydro-1 H-pyrazole-1-carbothioamide (D28)

Appearance: white powder**.** Melting Point**:** 236–237°C. ^1^ H NMR (300 MHz, DMSO-d6): 3.07–3.13 (dd, J1 = 4.18, J2 = 17.86 Hz, 1 H); 3.83–3.92 (dd, J1 = 11.51, J2 = 17.96 Hz, 1 H); 6.35–6.38 (dd, J1 = 4.36, J2 = 11.43 Hz, 1 H); 7.12–7.14 (m, 1 H); 7.19–7.26 (m, 3 H); 7.36–7.43 (m, 2 H); 7.51 (s, 1 H). MS (ESI): 455.0 (C16H13Br2FN3S, [M + H]^+^). Anal. Calcd for C16H12Br2FN3S: C, 42.04; H, 2.65; N, 9.19. Found: C, 42.16; H, 2.43; N, 9.31.

#### 5-(2-Chlorophenyl)-3-(3,4-dibromophenyl)-4,5-dihydro-1 H-pyrazole-1-carbothioamide (D29)

Appearance: white powder**.** Melting Point**:** 219–221°C. ^1^ H NMR (300 MHz, DMSO-d6): 3.08–3.12 (dd, J1 = 4.26, J2 = 17.92 Hz, 1 H); 3.89–3.95 (dd, J1 = 11.53, J2 = 17.91 Hz, 1 H); 6.33–6.35 (dd, J1 = 4.26, J2 = 11.52 Hz, 1 H); 7.04–7.07 (m, 1 H); 7.17–7.26 (m, 3 H); 7.36–7.44 (m, 2 H); 7.50 (s, 1 H). MS (ESI): 471.0 (C16H13Br2ClN3S, [M + H]^+^). Anal. Calcd for C16H12Br2ClN3S: C, 40.58; H, 2.55; N, 8.81. Found: C, 40.69; H, 2.38; N, 8.72.

#### 5-(2-Bromophenyl)-3-(3,4-dibromophenyl)-4,5-dihydro-1 H-pyrazole-1-carbothioamide (D30)

Appearance: white powder**.** Melting Point**:** 214–216°C. ^1^ H NMR (300 MHz, DMSO-d6): 3.09–3.15 (dd, J1 = 4.21, J2 = 17.91 Hz, 1 H); 3.84–3.91 (dd, J1 = 11.52, J2 = 17.93 Hz, 1 H); 6.29–6.32 (dd, J1 = 4.26, J2 = 11.51 Hz, 1 H); 7.06–7.09 (m, 1 H); 7.19–7.23 (m, 3 H); 7.34–7.45 (m, 2 H); 7.50 (s, 1 H). MS (ESI): 515.0 (C16H13Br3N3S, [M + H]^+^). Anal. Calcd for C16H12Br3N3S: C, 37.09; H, 2.33; N, 8.11. Found: C, 37.19; H, 2.42; N, 8.32.

#### General synthetic procedure of pyrazole derivatives F1–F16

A solution of chalcone (5 mmol) in 30 mL of acetic acid was added dropwise to 0.6 mL of hydrazine hydrate (12.5 mmol) and kept under stirring at 120°C for 24 h. The mixture was then poured onto ice-water, the crude pyrazole derivatives D1–D16 were obtained by vacuum filtration, which were crystallized from ethanol to give white powder compounds in good to excellent yields.

#### 1-(3-(3,4-Dichlorophenyl)-5-(4-fluorophenyl)-4,5-dihydro-1 H-pyrazol-1-yl)ethanone (F1)

Appearance: white powder**.**^1^HNMR (300 MHz, DMSO-d6): 2.30 (s, 3 H); 3.17–3.22 (dd, J1 = 2.91, J2 = 10.98 Hz, 1 H); 3.80–3.86 (dd, J1 = 7.32, J2 = 10.98 Hz, 1 H); 5.55– 5.58 (dd, J1 = 2.94, J2 = 7.14 Hz, 1 H); 7.12–7.16 (t, J = 5.31 Hz, 2 H); 7.22–7.25 (m, 2 H); 7.72–7.78 (m, 2 H); 7.97 (d, J = 11.10 Hz, 1 H). MS (ESI): 351.0 (C17H14Cl2FN2O, [M + H]^+^). Anal. Calcd for C17H13Cl2FN2O: C, 58.14; H, 3.73; N, 7.98. Found: C, 58.16; H, 3.71; N, 7.84.

#### 1-(5-(4-Chlorophenyl)-3-(3,4-dichlorophenyl)-4,5-dihydro-1 H-pyrazol-1-yl)ethanone (F2)

Appearance: white powder**.**^1^HNMR (300 MHz, DMSO-d6): 2.30 (s, 3 H); 3.17–3.22 (dd, J1 = 2.90, J2 = 10.96 Hz, 1 H); 3.81–3.87 (dd, J1 = 7.30, J2 = 10.91 Hz, 1 H); 5.54–5.57 (dd, J1 = 2.96, J2 = 7.14 Hz, 1 H); 7.21–7.23 (d, J = 5.13 Hz, 2 H); 7.37–7.39 (d, J = 4.95 Hz, 2 H); 7.72–7.78 (m, 2 H); 7.96–7.97 (d, J = 11.10 Hz, 1 H). MS (ESI): 367.0 (C17H14Cl3N2O, [M + H]^+^). Anal. Calcd for C17H13Cl3N2O: C, 55.54; H, 3.56; N, 7.62. Found: C, 55.52; H, 3.58; N, 7.53.

#### 1-(5-(4-Bromophenyl)-3-(3,4-dichlorophenyl)-4,5-dihydro-1 H-pyrazol-1-yl)ethanone (F3)

Appearance: white powder**.**^1^HNMR (300 MHz, DMSO-d6): 2.30 (s, 3 H); 3.17–3.22 (dd, J1 = 2.91, J2 = 10.96 Hz, 1 H); 3.81–3.87 (dd, J1 = 7.34, J2 = 10.98 Hz, 1 H); 5.52–5.56 (dd, J1 = 2.96, J2 = 7.17 Hz, 1 H); 7.21–7.23 (d, J = 5.13 Hz, 2 H); 7.37–7.39 (d, J = 4.95 Hz, 2 H); 7.72–7.78 (m, 2 H); 7.96–7.97 (d, J = 11.10 Hz, 1 H). MS (ESI): 410.9 (C17H14BrCl2N2O, [M + H]^+^). Anal. Calcd for C17H13BrCl2N2O: C, 49.55; H, 3.18; N, 6.80. Found: C, 49.57; H, 3.17; N, 6.68.

#### 1-(3-(3,4-Dichlorophenyl)-5-p-tolyl-4,5-dihydro-1 H-pyrazol-1-yl)ethanone (F4)

Appearance: white powder**.**^1^ H NMR (300 MHz, DMSO-d6): 2.26 (s, 3 H); 2.29 (s, 3 H); 3.13–3.17 (dd, J1 = 2.76, J2 = 10.80 Hz, 1 H); 3.79–3.85 (dd, J1 = 7.32, J2 = 10.98 Hz, 1 H); 5.49–5.52 (dd, J1 = 2.76, J2 = 7.14 Hz, 1 H); 7.05–7.13 (dd, J1 = 4.74, J2 = 17.37 Hz, 4 H); 7.71–7.77 (m, 2 H); 7.96–7.97 (d, J = 11.1 Hz, 1 H). MS (ESI): 347.1 (C18H17Cl2N2O, [M + H]^+^). Anal. Calcd for C18H16Cl2N2O: C, 62.26; H, 4.64; N, 8.07. Found: C, 62.27; H, 4.62; N, 8.21.

#### 1-(3-(3,4-Dichlorophenyl)-5-(4-methoxyphenyl)-4,5dihydro-1 H-pyrazol-1-yl)ethanone (F5)

Appearance: white powder**.**^1^ H NMR (300 MHz, DMSO-d6): 2.25 (s, 3 H); 3.11–3.16 (dd, J1 = 2.73, J2 = 10.80 Hz, 1 H); 3.68 (s, 3 H); 3.74–3.80 (dd, J1 = 7.14, J2 = 10.80 Hz, 1 H); 5.45–5.49 (dd, J1 = 2.73, J2 = 6.93 Hz, 1 H); 6.83– 6.85 (d, J = 5.31 Hz, 2 H); 7.07–7.08 (d, J = 5.31 Hz, 2 H); 7.69–7.70 (d, J = 5.13 Hz, 1 H); 7.73–7.75 (dd, J1 = 1.29, J2 = 5.13 Hz, 1 H); 7.94 (d, J = 11.1 Hz, 1 H). MS (ESI): 363.1 (C18H17Cl2N2O2, [M + H]^+^). Anal. Calcd for C18H16Cl2N2O2: C, 59.52; H, 4.44; N, 7.71. Found: C, 59.53; H, 4.42; N, 7.85.

#### 1-(5-(2-Chlorophenyl)-3-(3,4-dichlorophenyl)-4,5-dihydro-1 H-pyrazol-1-yl)ethanone (F6)

Appearance: white powder**.**^1^HNMR (300 MHz, DMSO-d6): 2.30 (s, 3 H); 3.15–3.24 (dd, J1 = 2.91, J2 = 10.96 Hz, 1 H); 3.82–3.89 (dd, J1 = 7.34, J2 = 10.98 Hz, 1 H); 5.51–5.58 (dd, J1 = 2.96, J2 = 7.17 Hz, 1 H); 7.21–7.25 (d, J = 5.13 Hz, 2 H); 7.35–7.39 (d, J = 4.95 Hz, 2 H); 7.72–7.78 (m, 2 H); 7.94–7.97 (d, J = 11.10 Hz, 1 H). MS (ESI): 367.0 (C17H14Cl3N2O, [M + H]^+^). Anal. Calcd for C17H13Cl3N2O: C, 55.54; H, 3.56; N, 7.62. Found: C, 55.56; H, 3.53; N, 7.78.

#### 1-(3-(3,4-Dichlorophenyl)-5-phenyl-4,5-dihydro-1Hpyrazol-1-yl)ethanone (F7)

Appearance: white powder**.**^1^ H NMR (300 MHz, DMSO-d6): 2.31 (s, 3 H); 3.14–3.22 (dd, J1 = 4.74, J2 = 18.27 Hz, 1 H); 3.79–3.89 (dd, J1 = 11.88, J2 = 18.30 Hz, 1 H); 5.53–5.58 (dd, J1 = 4.77, J2 = 11.91 Hz, 1 H); 7.17–7.35 (m, 5 H); 7.70–7.79 (m, 2 H); 7.70–7.72 (s, 1 H). MS (ESI): 333.1 (C17H15Cl2N2O, [M + H]^+^). Anal. Calcd for C17H14Cl2N2O: C, 61.28; H, 4.23; N, 8.41. Found: C, 61.26; H, 4.25; N, 8.56.

#### 1-(3-(3,4-Dichlorophenyl)-5-(3,5-dimethoxyphenyl)4,5-dihydro-1 H-pyrazol-1-yl)ethanone (F8)

Appearance: white powder**.**^1^ H NMR (300 MHz, DMSO-d6): 2.26 (s, 3 H); 3.12–3.16 (dd, J1 = 2.73, J2 = 10.80 Hz, 1 H); 3.68 (s, 6 H); 3.73–3.83 (dd, J1 = 7.14, J2 = 10.80 Hz, 1 H); 5.45–5.49 (dd, J1 = 2.73, J2 = 6.93 Hz, 1 H); 6.81– 6.85 (d, J = 5.31 Hz, 2 H); 7.06–7.08 (d, J = 5.31 Hz, 2 H); 7.69–7.70 (d, J = 5.13 Hz, 1 H); 7.72–7.75 (dd, J1 = 1.29, J2 = 5.13 Hz, 1 H); 7.95 (d, J = 11.1 Hz, 1 H). MS (ESI): 393.1 (C19H19Cl2N2O3, [M + H]^+^). Anal. Calcd for C19H18Cl2N2O3: C, 58.03; H, 4.61; N, 7.12. Found: C, 58.05; H, 4.59; N, 7.26.

#### 1-(3-(3,4-Dimethylphenyl)-5-(4-fluorophenyl)-4,5-dihydro-1 H-pyrazol-1-yl)ethanone (F9)

Appearance: white powder**.**^1^ H NMR (300 MHz, DMSO-d6): 2.25 (s, 6 H); 2.29 (s, 3 H); 3.07–3.14 (dd, J1 = 2.76, J2 = 10.86 Hz, 1 H); 3.76–3.86 (dd, J1 = 7.32, J2 = 10.80 Hz, 1 H); 5.50–5.54 (dd, J1 = 2.76, J2 = 6.96 Hz, 1 H); 7.09–7.23 (m, 5 H); 7.48–7.50 (d, J = 7.86, 1 H); 7.56 (s, 1 H); MS (ESI): 311.2 (C19H20FN2O, [M + H]^+^). Anal. Calcd for C19H19FN2O: C, 73.53; H, 6.17; N, 9.03. Found: C, 73.55; H, 6.16; N, 9.16.

#### 1-(5-(4-Chlorophenyl)-3-(3,4-dimethylphenyl)-4,5-dihydro-1 H-pyrazol-1-yl)ethanone (F10)

Appearance: white powder**.**^1^ H NMR (300 MHz, DMSO-d6): 2.25 (s, 6 H); 2.29 (s, 3 H); 3.06–3.14 (dd, J1 = 2.76, J2 = 10.86 Hz, 1 H); 3.77–3.87 (dd, J1 = 7.32, J2 = 10.80 Hz, 1 H); 5.49–5.55 (dd, J1 = 2.76, J2 = 6.96 Hz, 1 H); 7.18–7.23 (m, 3 H); 7.36–7.39 (d, J = 8.22, 2 H); 7.47–7.50 (d, J = 8.04, 2 H); 7.56 (s, 1 H); MS (ESI): 327.1 (C19H20ClN2O, [M + H]^+^). Anal. Calcd for C19H19ClN2O: C, 69.83; H, 5.86; N, 8.57. Found: C, 69.84; H, 5.84; N, 8.64.

#### 1-(5-(4-Bromophenyl)-3-(3,4-dimethylphenyl)-4,5-dihydro-1 H-pyrazol-1-yl)ethanone (F11)

Appearance: white powder**.**^1^ H NMR (300 MHz, DMSO-d6): 2.25 (s, 6 H); 2.29 (s, 3 H); 3.07–3.14 (dd, J1 = 4.56, J2 = 18.09 Hz, 1 H); 3.76–3.86 (dd, J1 = 11.88, J2 = 18.09 Hz, 1 H); 5.47–5.53 (dd, J1 = 4.38, J2 = 11.70 Hz, 1 H); 7.12–7.15 (d, J = 8.43, 2 H); 7.20–7.23 (d, J = 7.86, 1 H); 7.47–7.55 (m, 4 H). MS (ESI): 371.1 (C19H20BrN2O, [M + H]^+^). Anal. Calcd for C19H19BrN2O: C, 61.47; H, 5.16; N, 7.55. Found: C, 61.49; H, 5.13; N, 7.69.

#### 1-(3-(3,4-Dimethylphenyl)-5-p-tolyl-4,5-dihydro-1Hpyrazol-1-yl)ethanone (F12)

Appearance: white powder**.**^1^ H NMR (300 MHz, DMSO-d6): 2.22 (s, 6 H); 2.25 (s, 6 H); 3.01–3.06 (dd, J1 = 2.73, J2 = 10.80 Hz, 1 H); 3.73–3.79 (dd, J1 = 6.96, J2 = 10.80 Hz, 1 H); 5.42–5.46 (dd, J1 = 2.55, J2 = 8.14 Hz, 1 H); 7.01–7.02 (d, J = 4.77, 2 H); 7.07–7.09 (d, J = 4.74, 2 H); 7.17–7.19 (d, J = 4.59, 1 H);7.45– 7.46 (d, J = 4.74, 1 H); 7.53 (s, 1 H). MS (ESI): 307.2 (C20H23N2O, [M + H]^+^). Anal. Calcd for C20H22N2O: C, 78.40; H, 7.24; N, 9.14. Found: C, 78.43; H, 7.22; N, 9.25.

#### 1-(3-(3,4-Dimethylphenyl)-5-(4-methoxyphenyl)-4,5dihydro-1 H-pyrazol-1-yl)ethanone (F13)

Appearance: white powder**.**^1^ H NMR (300 MHz, DMSO-d6): 2.25 (s, 6 H); 2.27 (s, 3 H); 3.04–3.11 (dd, J1 = 3.84, J2 = 12.09 Hz, 1 H); 3.71 (s, 3 H); 3.76–3.82 (dd, J1 = 11.68, J2 = 18.06 Hz, 1 H); 5.44–5.47 (dd, J1 = 4.26, J2 = 10.91 Hz, 1 H); 6.85–6.87 (d, J = 7.24, 2 H); 7.07–7.10 (d, J = 7.22, 2 H); 7.20– 7.22 (d, J = 4.86, 2 H); 7.47–7.56 (m, 2 H). MS (ESI): 323.2 (C20H23N2O2, [M + H]^+^). Anal. Calcd for C20H22N2O2: C, 74.51; H, 6.88; N, 8.69. Found: C, 74.53; H, 6.85; N, 8.76.

#### 1-(5-(2-Chlorophenyl)-3-(3,4-dimethylphenyl)-4,5dihydro-1 H-pyrazol-1-yl)ethanone (F14)

Appearance: white powder**.**^1^ H NMR (300 MHz, DMSO-d6): 2.24 (s, 6 H); 2.34 (s, 3 H); 2.98–3.06 (dd, J1 = 4.74, J2 = 17.91 Hz, 1 H); 3.86–3.96 (dd, J1 = 11.88, J2 = 17.91 Hz, 1 H); 5.70–5.76 (dd, J1 = 4.59, J2 = 11.73 Hz, 1 H); 7.02–7.05 (m, 1 H); 7.19–7.22 (d, J = 7.86 Hz, 1 H); 7.27–7.30 (m, 2 H); 7.47–7.55 (m, 3 H). MS (ESI): 327.1 (C19H20ClN2O, [M + H]^+^). Anal. Calcd for C_19_H_19_ClN_2_O: C, 69.83; H, 5.86; N, 8.57. Found: C, 69.85; H, 5.84; N, 8.71.

#### 1-(3-(3,4-Dimethylphenyl)-5-phenyl-4,5-dihydro-1Hpyrazol-1-yl)ethanone (F15)

Appearance: white powder**.**^1^ H NMR (300 MHz, DMSO-d6): 2.25 (s, 6 H); 2.30 (s, 3 H); 3.05–3.12 (dd, J1 = 4.38, J2 = 18.12 Hz, 1 H); 3.77–3.87 (dd, J1 = 11.91, J2 = 18.12 Hz, 1 H); 5.49–5.54 (dd, J1 = 4.23, J2 = 11.73 Hz, 1 H); 7.15–7.34 (m, 6 H); 7.48–7.56 (m, 2 H). MS (ESI): 293.2 (C19H21N2O, [M + H]^+^). Anal. Calcd for C_19_H_20_N_2_O: C, 78.05; H, 6.89; N, 9.58. Found: C, 78.07; H, 6.87; N, 9.67.

#### 1-(5-(3,5-Dimethoxyphenyl)-3-(3,4-dimethylphenyl)4,5-dihydro-1 H-pyrazol-1-yl)ethanone (F16)

Appearance: white powder**.**^1^ H NMR (300 MHz, DMSO-d6): 2.26 (s, 6 H); 2.27 (s, 3 H); 3.02–3.10 (dd, J1 = 3.84, J2 = 12.09 Hz, 1 H); 3.71 (s, 6 H); 3.74–3.81 (dd, J1 = 11.68, J2 = 18.06 Hz, 1 H); 5.42–5.47 (dd, J1 = 4.26, J2 = 10.91 Hz, 1 H); 6.84–6.87 (d, J = 7.24, 2 H); 7.06–7.10 (d, J = 7.22, 2 H); 7.20– 7.22 (d, J = 4.86, 2 H); 7.45–7.56 (m, 2 H). MS (ESI): 353.2 (C21H25N2O3, [M + H]^+^); Anal. Calcd for C_21_H_24_N_2_O_3_: C, 71.57; H, 6.86; N, 7.95. Found: C, 71.55; H, 6.87; N, 7.84.

### Anti-tumor cytotoxicity bioassay in vitro

#### In vitro antitumor assay

All chemicals and reagents are supplied from Sigma-Aldrich (SIGMA-ALDRICH Chemie GmbH, Steinheim, Germany) [29]. Animal house and biochemical equipments have been made available by the Cairo University, Egypt. Female Swiss albino mice weighing 25–30 g were used in this study (The Holding Company for Biological Products and Vaccines National Cancer institute, Cairo, Egypt. Mice were housed at a constant temperature (24 ± 2°C) with alternating 12 h- light and dark cycles and fed standard laboratory food and water. Tests were made in consideration of the internationally valid guidelines. The Medical Centre for Research, National Cancer Institute, Cairo, Egypt is concerned with biological and animal studies which have an approval of an institution responsible for animal ethics.

#### Cell growth inhibition assay

The *in-vitro* growth inhibitory activity of the test compounds against EAC cell line was evaluated in NCI. The evaluation depends on using the standard 48 h exposure assay [30]. Ehrlich Ascites Carcinoma (EAC) cells were obtained by needle aspiration of ascetic fluid from the pre-inoculated mice under aseptic conditions. Tumor cells suspension (2.5 × 10^6^ per ml) was prepared in saline. The parent line was kindly supplied by the National Cancer institute (NCI), Cairo, Egypt, Diagnostics Lab. Cairo University, Egypt. The tumor cells were maintained by weekly intra-peritoneal transplantation of cells.

#### Bioassay in vitro

EAC (Ehrlich Ascites Carcinoma cell line) was obtained from the Pharmacology Unit, Cancer Biology Department, National Cancer Institute, Cairo University, Egypt. Cells were maintained in DMEM medium with 10% foetal calf serum, sodium pyruvate, 100 U/ml penicillin and 100 mg/ml streptomycin at 37^ο^C and 5% CO_2_. Potential cytotoxicity of D1-30 and F1-16 were tested using the method of Skeha et al. [31,32] briefly, 104 cells/well were plated onto 96-well dishes overnight before the treatment with the tested compounds to allow the attachment of cells to the wall of the plate. Different concentrations of each tested compound (0.1, 2.5, 5, 10 mg/ml) were added to the cell monolayer; triplicate wells were used for each individual dose. Monolayer cells were incubated with the tested agent(s) for 48 h at 37°C and 5% CO_2_. At the end of the incubation period, the cells were fixed and stained with sulforhodamine B dissolved in acetic acid. Unbound stain was removed by washing four times with 1% acetic acid and the protein bound dye was extracted with tris–EDTA buffer. Absorbance was measured in an ELISA reader. The relation between surviving fraction and compound concentration was plotted to get the survival curve of each tumor cell line and IC_50_, the concentration of an agent that causes a 50% growth inhibition for each tested agent using each cell line was obtained from the survival curve.

## Competing interests

The authors declare that they have no competing interests.

## Authors’ contributions

ASE developed the study concept and aims and initiated the project. All authors assisted in further development of the protocol. ASE and SRA-A were responsible for drafting the manuscript. ASSHE, DHSS, SRA-A, DAI implement the protocol and oversee collection of the data. All authors contributed to the final manuscript.

## Disclosure

The authors report no conflicts of interest in this work.
